# Shionone Attenuates Sepsis-Induced Acute Kidney Injury by Regulating Macrophage Polarization *via* the ECM1/STAT5 Pathway

**DOI:** 10.3389/fmed.2021.796743

**Published:** 2022-01-24

**Authors:** Biao Zhang, Yi Xue, Jin Zhao, Huojun Jiang, Jiaoli Zhu, Hao Yin, Yizhen Qiu, Aihao Hu, Lingqi Xu, Yi Song, Xin Wang

**Affiliations:** ^1^Department of Critical Care Medicine, Suzhou Hospital of Integrated Traditional Chinese and Western Medicine, Suzhou, China; ^2^Department of Nephrology, Suzhou Hospital of Integrated Traditional Chinese and Western Medicine, Suzhou, China; ^3^Li Shicai School Inheritance Studio, Suzhou Hospital of Integrated Traditional and Western Medicine, Suzhou, China

**Keywords:** acute kidney injury, ECM1, macrophages, inflammation, shionone

## Abstract

**Backgrounds:**

To date, there are no specific drugs approved for the treatment of sepsis associated acute kidney injury (AKI). Shionone is a natural component with anti-inflammatory activity. In this study, we sought to determine the functional role of Shionone in sepsis-induced AKI.

**Methods:**

Animal models of AKI were constructed by cecum ligation and puncture (CLP) surgery. C57BL/6 mice were randomly assigned to the Sham, CLP, 10 mg/kg DXM, 50 mg/kg Shionone and 100 mg/kg Shionone groups. RAW264.7 treated with lipopolysaccharides (LPS) was used as an *in vitro* sepsis model and cells were divided into control, LPS, 1 μg/mL Shionone and 2 μg/mL Shionone groups. The pathological status was assessed by Hematoxylin-Eosin (HE) staining assay, protein expressions were detected by immunofluorescence staining and Western blot, macrophage typing was detected by flow, and the levels of pro-inflammatory factors (IL-6, IL-12, IL-1β, TNF-α) and anti-inflammatory factors (IL-10 and TGF-β) were measured using the corresponding kits.

**Results:**

ECM1 is highly expressed in tissue-infiltrating macrophages under inflammatory conditions. It has been observed that Shionone inhibits the expression of ECM1 and attenuates sepsis-induced injury in kidney and inflammatory factor levels in serum. In addition, Shionone may reduce inflammatory factor levels through the promotion of M2 macrophages by GM-CSF/STAT5/Arg1 pathway to alleviate sepsis induced inflammation *in vitro*.

**Conclusion:**

These findings demonstrate that Shionone can alleviate sepsis-induced AKI by promoting M2 macrophage polarization through regulating the ECM1/STAT5 pathway.

## Introduction

Sepsis is a syndrome of systemic inflammatory response caused by the invasion of pathogenic microorganisms into the body and resulting in extensive cellular and organismal injury (1). Acute kidney injury (AKI) induced by sepsis is the most common complication in critically ill patients. Within hours or days after the onset of sepsis, kidney function declines rapidly, leading to elevated serum creatinine levels (2). An effective treatment for AKI has still not been found, and it is therefore important to clarify the mechanism of AKI for its treatment.

Clinical treatment of sepsis associated AKI is mainly based on renal replacement therapy with drugs such as antibiotics and glucocorticoids, but is prone to drug resistance and risks of immunosuppression, making clinical treatment increasingly difficult (3, 4). The disruption of the inflammatory response in sepsis is closely linked to the massive activation of immune cells and the overproduction of pro-inflammatory factors, and increased inflammation can lead to multi-organ dysfunction and death (5, 6).

Macrophages are an important component of innate and adaptive immunity and play an important role in the regulation of the inflammatory response in sepsis (7). Macrophages are polarized into two phenotypes, type M1 (classically activated) and type M2 (alternatively activated), which regulate immune homeostasis in the organism (8). M1 macrophages release large amounts of pro-inflammatory factors such as IL-1, tumor necrosis factor-α (TNF-α) and IL-6 to eliminate host pathogens (9, 10); M2 macrophages stimulate large amounts of the inflammation-suppressing factor IL-10 growth factor-β (TGF-β) and other anti-inflammatory cytokines (11). In a systemic inflammatory response, some macrophages are aberrantly recruited and continue to be activated, ultimately leading to acute organ dysfunction. Extracellular Matrix Protein 1 (ECM1) is highly expressed in macrophages, particularly in inflammatory tissue infiltrated macrophages (12). It has been shown that ECM1 can regulate M1 macrophage polarization *via* granulocyte-macrophage colony-stimulating factor (GM-CSF) (12). In response to LPS stimulation, GM-CSF expression is increased, thereby reducing the expression of pro-inflammatory cytokines (12, 13).

Shionone is a natural component extracted from the dried rhizome of *Aster tataricus* L. f. with anti-inflammatory effects (14, 15). Previous studies have shown that the development of sepsis is associated with stimulation of LPS and that injection of LPS into animals or treatment of LPS to cells activates an inflammatory response similar to that characterized by clinical sepsis (12, 13). In the present study, we hypothesized that Shionone may protect renal function in sepsis-induced AKI by modulating macrophage inflammation and we validated our hypothesis using a successfully established a mouse model of sepsis-induced AKI and LPS-stimulated RAW264.7.

## Materials and Methods

### Reagents and Antibodies

Antibodies used for this study were shown below: Arg1 (93668, CST), CD16/CD32 (ab228971, Abcam), CD26 (ab187048, Abcam), ECM1 (ab126629, Abcam), iNOS (ab178945, Abcam), p-NF-κB (3033, CST), NF-κB (8242, CST), p-STAT5 (ab98338, Abcam), and STAT5 (ab230670, Abcam). Elisa kits for the detection of pro-inflammatory factors IL-6 (ZC-36404), IL-12 (ZC-36323), IL-1β (ZC-36391), TNF-α (ZC-37624), and anti-inflammatory factors IL-10 (ZC-36379), TGF-β (ZC-37644) purchased from ZCIBIO Technology Co., Ltd. Shionone (S823560, purity ≥ 98%) was purchased from Macklin Inc.

### Animals and Experimental Protocol

C57BL/6 mice were purchased from Jiangsu ALF Biotechnology Co. LTD. They were housed at 22 ± 1°C with 12-light/dark cycle and were allowed to drink and eat freely. After 1 week of temporary housing, mice were modeled for AKI using cecum ligation and puncture (CLP) surgery (16). In the CLP model, sepsis originates as a microbial infectious lesion in the abdominal cavity, follow by transfer of bacteria into the bloodstream, which then triggers a systemic inflammatory response. Mice received CLP surgery were randomly divided into four groups (*n* = 12 in each group): CLP model group, 10 mg/kg Dexamethasone (DXM) group, 50 mg/kg-Shionone group and 100 mg/kg Shionone group. They were resuscitated at the end of CLP surgery and the mice in the sham group were only opened and not ligated in the abdominal cavity. Shionone and DXM were administered at −2, 0, 2, and 12 h after CLP, and samples were taken 24 h post-CLP. All animal experiments were approved by the Institutional Animal Care and Use Committee of Suzhou Hospital of Integrated Traditional Chinese and Western Medicine.

### Cells Culture and Treatment

Mouse macrophages RAW264.7 were obtained from Nanjing University of Chinese Medicine. RAW264.7 cells were cultured in DMEM high sugar medium containing 10% fatal bovine serum (FBS) and 100 U/mL penicillin and streptomycin in an incubator set to 5% CO_2_ at 37°C. Cells were incubated with different concentrations of LPS, and depending on cell viability, we chose 5 μg/mL of LPS co-incubated with cells for 24 h to establish the model. After treatment of LPS, cells were collected for subsequent experiments.

### Cell Viability Assay

Cell viability was assayed using the MTT method according to the previous study (17). In brief, cultured cells were incubated with a gradient concentration of Shionone at 37°C for 24 h. Ten microliter of MTT solution was added to each well and incubation continued for 4 h. The incubation was terminated and the crystals were dissolved in DMSO. Absorbance was measured at 490 nm and using a microplate reader.

### Hematoxylin-Eosin Staining

HE staining method was used to detect the pathological morphology of the kidney tissue. Paraffin-embedded sections were stained with hematoxylin solution for 5 min to give a violet-blue color to the chromatin in the nucleus and ribosomes in the cytoplasm. The sections were stained with 0.5% eosin solution for 2 min to give a red color to the cytoplasmic and extracellular matrix components. Finally, the slides were sealed with resin and placed under a microscope for observation and photography. A double-blind method was used to evaluate tubular necrosis in mice for scoring (18), and the scoring criteria were: 0 score as normal, 1 score as mildly damaged (damaged tubules <5%), 2 score as mildly damaged (damaged tubules 5–25%), 3 score as moderately damaged (damaged tubules 25–75%) and 4 score as severely damaged (damaged tubules > 75%). Semi-quantitative analysis was performed and the mean value was calculated as an index to evaluate the degree of tubular necrosis.

### Immunofluoresence Staining

Immunofluorescence staining was performed as described in previous studies. The fixed cells were permeabilized using paraformaldehyde and then blocked for 30 min. Samples were incubated with CD16/CD32 Rat mAb (80366, CST) and Anti-Mannose Receptor antibody (ab64693, abcam) overnight at 4°C, followed by incubation with DAPI for 5 min. Finally, images were visualized and photographed using fluorescence microscopy.

### Detection of Biochemical Indicators in Blood Serum

Serum levels of IL-6, IL-12, IL-1β, TNF-α, and IL-10, TGF-β in mice were analyzed according to the manufacturer's instructions using commercial kits.

### Western Blot Assay

Kidney tissues and cells were lysed in RIPA buffer and then the contents of proteins were determined by a BCA protein assay kit (P0012S, Beyotime). Equal amounts of protein extracts were separated on 10% SDS-PAGE gels and transferred to polyethylene fluoride membranes. After serum closure, membranes were incubated with primary antibodies overnight at 4°C and then incubated with the corresponding secondary antibodies for 2 h at room temperature. Quantification of protein bands was analyzed using Image J software.

### Flow Detection of Macrophage Fractionation

Cells were collected and resuspended in 100 μL of PBS for 5 × 10^5^ cells/tube, then added CD16/32 antibody (ab1223200, Abcam) and PE-anti-mouse CD206 (141706, Biolegend) antibody and incubated for 30 min at 4°C. The supernatant was removed by centrifugation, Goat-anti-Rabbit IgG H&L (Alexa Flour 488) was added and incubated for 30 min at 4°C. Cells were then resuspended in 0.3 mL PBS and CD16/32 and CD206 expression was detected in a FACSCalibur flow cytometer.

### Statistical Analysis

The measurements are expressed as mean ± standard deviation (S.D.) and statistical analysis of the statistical significance of the differences was performed by independent *t*-tests. All analyses were performed using Graphpad Prism software. *p* < 0.05 was considered statistically significant.

## Results

### Shionone Ameliorates Renal Injury of Sepsis-Induced AKI in Mice

**Figure 1:**
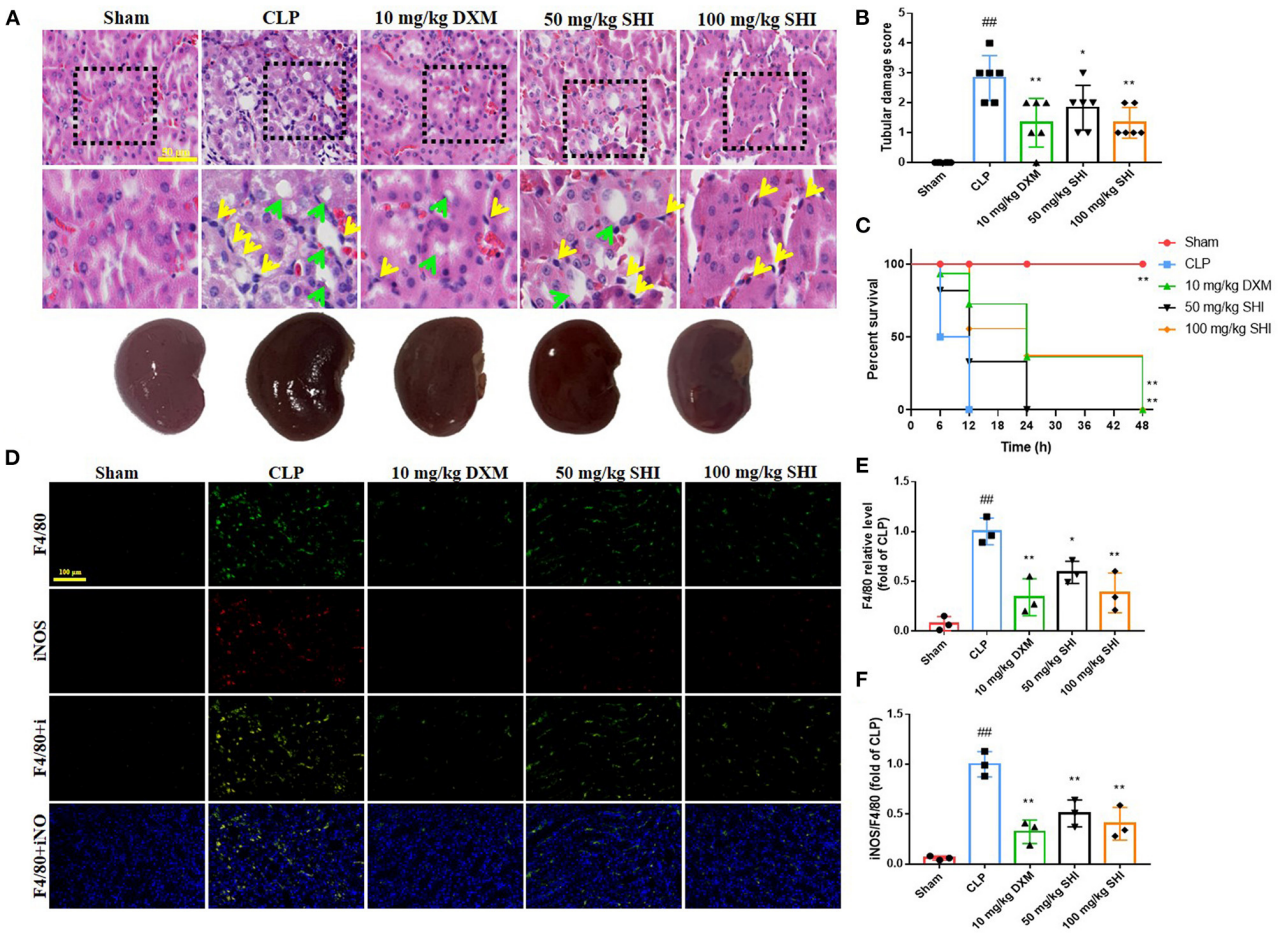
The effects of Shionone on sepsis-induced acute kidney injury model rats. **(A)** Kidney was collected 24 h after the stimulus for the evaluation of histopathological changes using HE staining, yellow arrow indicate inflammatory cell infiltration and green arrow indicate vacuolar degeneration (*n* = 3). **(B)** The tubular score of each group (*n* = 3). **(C)** The survival percentage of mice in each group (*n* = 6). **(D)** Representative fluorescent picture of F4/80 (biomarkers of macrophages) and iNOS (biomarkers of M1 type macrophages) of kidney in sham and kidney injury groups (*n* = 3). **(E)** Relative fluorescence of F4/80 of kidney in sham and kidney injury groups (*n* = 3). **(F)** Relative fluorescence of iNOS of kidney in sham and kidney injury groups (*n* = 3). Sham group was used as a negative control and CLP group as a positive control. Results are expressed as ± SD for independent experiments, ^##^*p* < 0.01 vs. Sham group, and *p < 0.05 vs. CLP group, **p < 0.01 vs. CLP group, one ANOVA followed by Tukey's *post-hoc*.

In Figure 1A, HE staining results showed that the glomeruli in the Sham group were complete and neatly structured, whereas the CLP model group had a large inflammatory cell infiltration (yellow arrows) and vacuolar degeneration (green arrows); inflammatory cell infiltration and vacuolation were reduced in the 50-Shionone, 100-Shionone and DXM groups compared with the CLP group, but 100-Shionone was better than DXM in improving the changes in vacuolar degeneration. Based on the score, it is also clear that Shionone significantly improved the pathological changes in AKI renal tubules (Figure 1B). Shionone and DXM also improved the survival percentage of CLP-induced rats (Figure 1C). F4/80 was a marker for macrophages and iNOS was a marker for M1-type macrophages. Combined with Figures 1D–F, the macrophage infiltration and the proportion of M1-type macrophages was significantly higher in the model group compared with the Sham group and they were decreased in the 50-Shionone, 100-Shionone and DXM groups, and the effects of 100-Shionone and DXM were comparable.

### Shionone Improves Serum and Kidney Biochemical Parameters of Sepsis-Induced AKI in Mice

**Figure 2:**
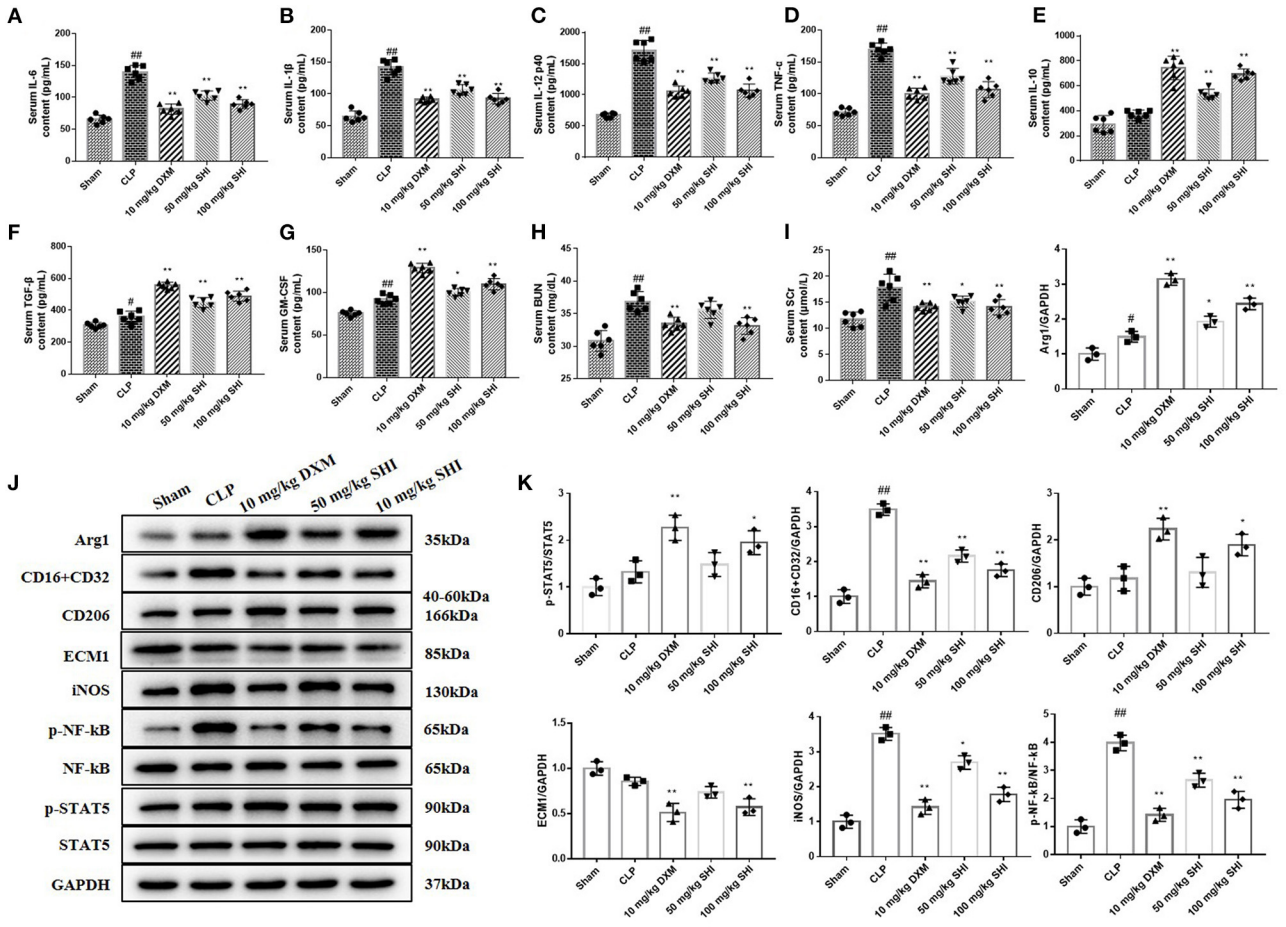
Improvement of serum and kidney biochemical parameters by Shionone in sepsis-induced acute kidney injury rats. **(A–G)** ELISA for the detection of inflammatory factors (IL-6, IL-1β, IL-12, TNF-α, IL-10, TGF-β, and GM-CSF) in the serum of sham and kidney injury groups (*n* = 6). **(H,I)** The contents of BUN and Scr in rats' serum in sham and kidney injury groups (*n* = 6). **(J)** Western blot analyses of renal expressions of Arg1, CD16+CD32, CD26, ECM1, iNOS, p-NF-κB, NF-κB, p-STAT5, and STAT5 in sham control and injured kidneys groups. **(K)** Relative expression analysis of Arg1, CD16+CD32, CD26, ECM1, iNOS, p-NF-κB, NF-κB, p-STAT5, and STAT5 (*n* = 3). Sham group was used as a negative control and CLP group as a positive control. Results are expressed as ± SD for independent experiments, ^#^*p* < 0.05 vs. Sham group, ^##^*p* < 0.01 vs. Sham group, and *p < 0.05 vs. CLP group, **p < 0.01 vs. CLP group, one ANOVA followed by Tukey's *post-hoc*.

Sepsis is characterized by the excessive release of inflammatory mediators (19). In this study, we detected the content of pro-inflammatory factors IL-6, IL-1β, IL-12, and TNF-α and anti-inflammatory factors IL-10 and TGF-β in serum. GM-CSF can reduce the expression of pro-inflammatory cytokines (20). With the treatment of Shionone and DXM, the contents of IL-6, IL-1β, IL-12, and TNF-α were decreased significantly compared with the CLP group, and the contents of IL-10, TGF-β, and GM-CSF were increased significantly (Figures 2A–G). Serum creatinine (SCr) and blood urea nitrogen (BUN) are indicators of renal function. We found that CLP modeling increased the levels of SCr and BUN, which were significantly reduced by the use of Shionone and DXM (Figures 2H,I, *p* < 0.05). The expressions of CD16/32, iNOS and p-NF-kB/NF-kB were increased in the CLP group than the Sham group, while Shionone and DXM reduced the increase in sepsis-induced AKI mice. The expressions of Arg1, p-STAT5/STAT5, and CD206 were increased with the treatment of Shionone and DXM compared to the CLP group, and the expression of ECM1 was reduced significantly with Shionone of 100 mg/kg and DXM of 10 mg/kg (Figures 2J,K).

### Shionone Protects RAW264.7 Cells From LPS Stimulation

**Figure 3:**
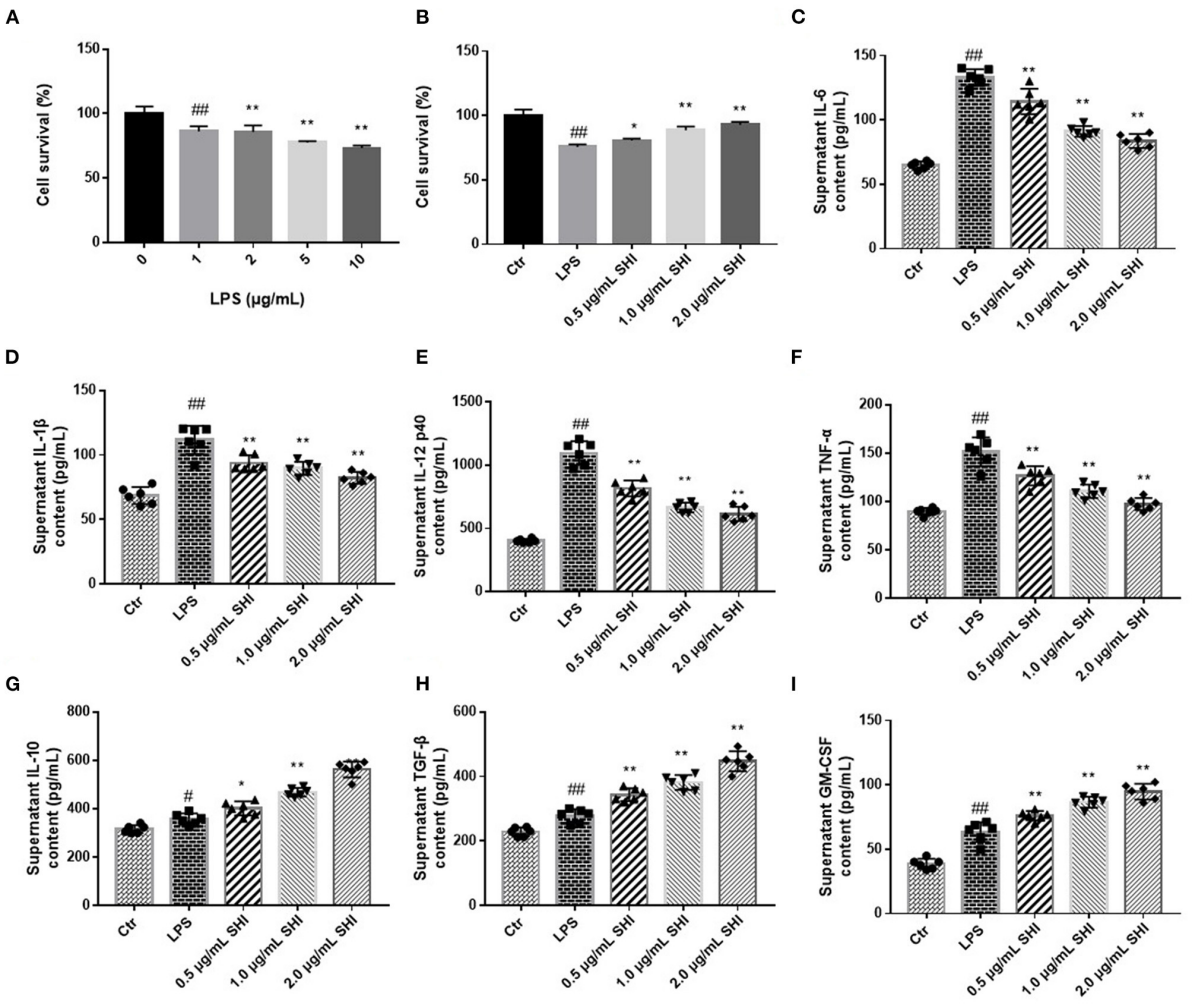
Shionone protects RAW264.7 cells from LPS stimulation. **(A)** LPS concentration screening for cellular modeling (*n* = 3). **(B)** Survival rate of RAW264.7 cells with 0.5, 1, and 2 μg/mL Shionone (*n* = 3). **(C–I)** ELISA to detect inflammatory factors (IL-6, IL-1β, IL-12, TNF-α, IL-10, TGF-β, and GM-CSF) in LPS-treated RAW264.7 cells' supernatants (*n* = 6). Control group was used as a negative control and LPS group as a positive control. Results are expressed as ± SD for independent experiments, ^#^*p* < 0.05 vs. Control group, ^##^*p* < 0.01 vs. LPS group, and *p < 0.05 vs. Control group, **p < 0.01 vs. LPS group, one ANOVA followed by Tukey's *post-hoc*.

We incubated the cells with different concentrations of LPS and assayed their viability, as shown in Figure 3A, and 5 μg/mL of LPS was selected as the modeling concentration. In Figure 3B, the protective effect of Shionone on cell viability can be observed, and the viability of the 2 μg/mL Shionone group was higher than that of the 0.5 μg/mL Shionone and 1 μg/mL Shionone groups. Shionone reduced the contents of IL-6, IL-1β, IL-12, and TNF-α and increased the contents of IL-10, TGF-β, and GM-CSF in a dose-dependent manner (Figures 3C–I).

### Shionone Regulates Macrophage-Dependent Inflammation

**Figure 4:**
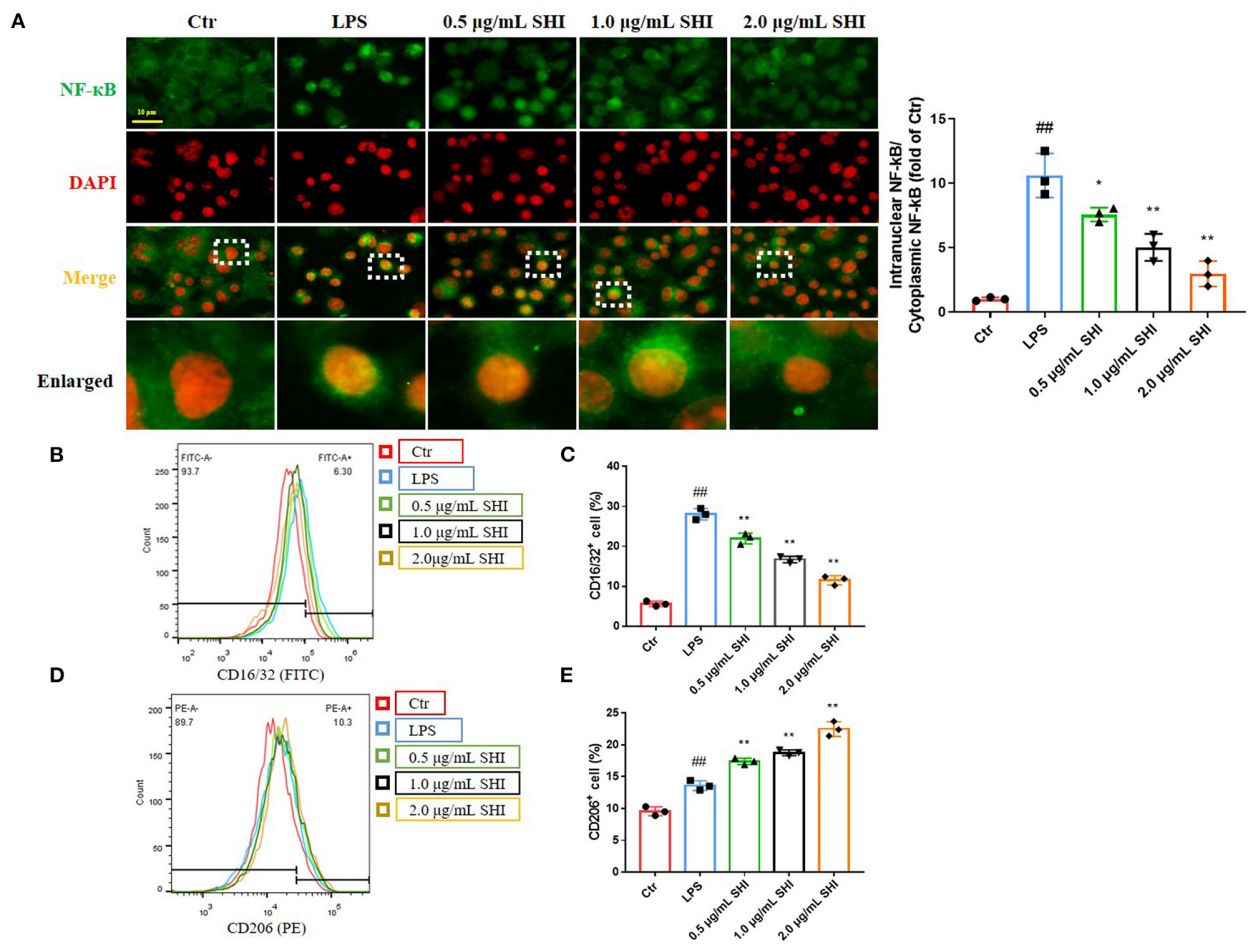
The effect of Shionone on macrophage inflammation. **(A)** Representative images of NF-κB in nucleus and cytoplasm detected by immunofluorescence (*n* = 3). **(B,C)** CD16/32 flow assay for M1 macrophages and the proportion of M1 macrophages in each group (*n* = 3). **(D,E)** CD206 flow assay for M2 macrophages and the proportion of M2 macrophages in each group (*n* = 3). Control group was used as a negative control and LPS group as a positive control. Results are expressed as ± SD for independent experiments, ^##^*p* < 0.01 vs. LPS group, and *p < 0.05 vs. Control group, **p < 0.01 vs. LPS group, one ANOVA followed by Tukey's *post-hoc*.

We used immunofluorescence to detect NF-κB activation into the nucleus and nucleus was stained by DAPI. As shown in Figure 4A, the nuclear translocation of NF-κB was increased following LPS treatment, as indicated by increased intensity of NF-κB in the nucleus and the increased nucleus to cytoplasmic intensity ratio of NF-κB. Whereas, shionone treatment resulted in a decrease of the cytoplasm to nucleus translocation of NF-κB in a dose-dependent manner that was evident from the reduction of the nucleus to cytoplasmic intensity ratio of NF-κB. CD16/32 levels increased after LPS modeling and reduced by the treatment of Shionone, while CD206 levels increased slightly after LPS treatment and further after Shionone use (Figures 4B–E). This suggested that Shionone could inhibit M1 macrophages and increase M2 macrophages in the treatment of LPS *in vitro* model.

### The Effects of Shionone on Arg1, CD16+CD32, CD26, ECM1, INOS, p-NF-κB, NF-κB, p-STAT5, and STAT5 in LPS-Treated RAW264.7 Cells

**Figure 5:**
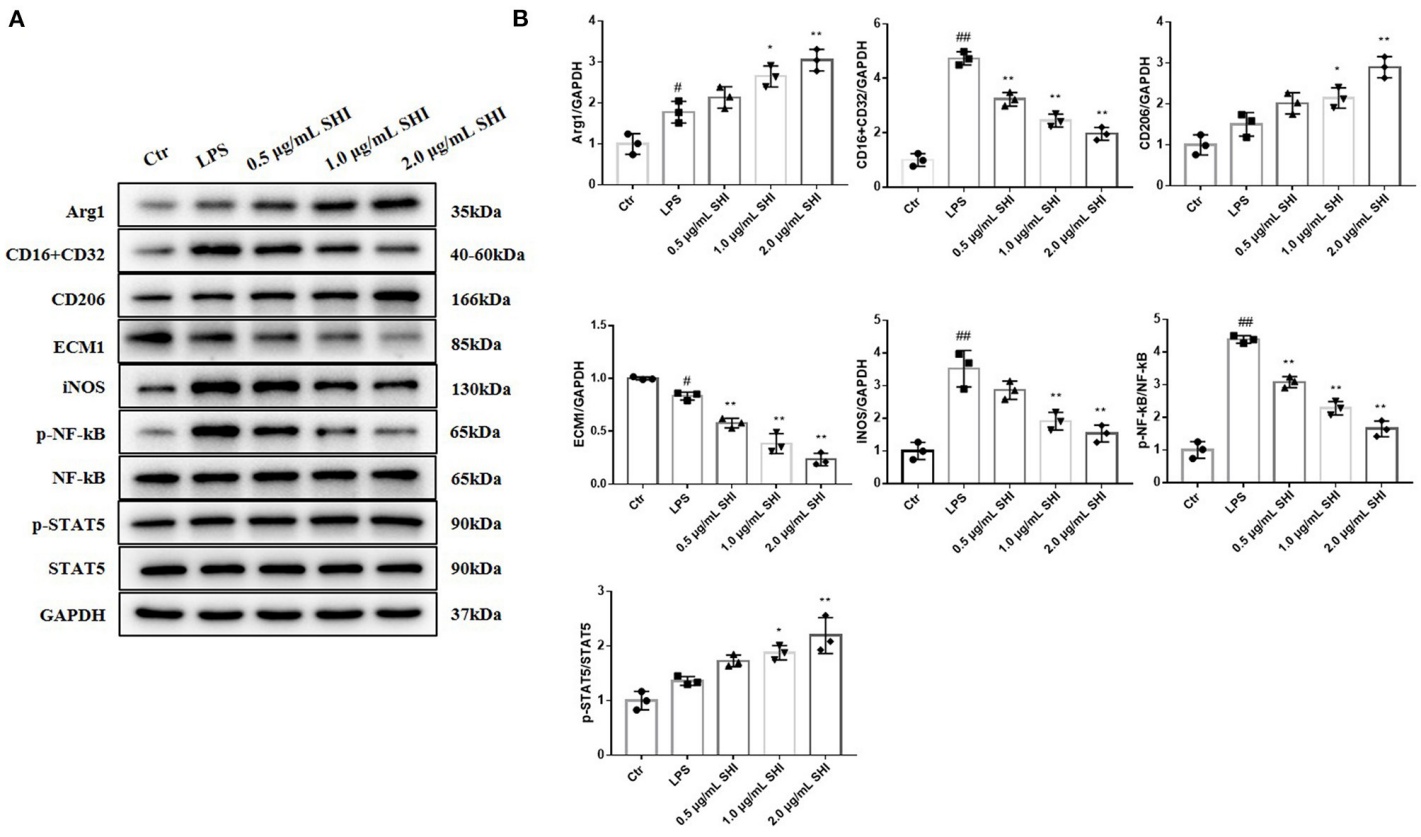
The effects of Shionone on Arg1, CD16+CD32, CD26, ECM1, iNOS, p-NF-κB, NF-κB, p-STAT5, and STAT5 in LPS-treated RAW264.7 cells. **(A)** Western blot analyses of expressions of Arg1, CD16+CD32, CD26, ECM1, iNOS, p-NF-κB, NF-κB, p-STAT5, and STAT5 in LPS-stimulated RAW264.7 cells with or without Shionone treatment (*n* = 3). **(B)** Histogram of relative expression of each protein (*n* = 3). Control group was used as a negative control and LPS group as a positive control. Results are expressed as ± SD for independent experiments, ^#^*p* < 0.05 vs. Control group, ^##^*p* < 0.01 vs. LPS group, and *p < 0.05 vs. Control group, **p < 0.01 vs. LPS group, one ANOVA followed by Tukey's *post-hoc*.

The expressions of CD16/32, iNOS, and p-NF-κB/NF-κB were increased in the LPS group than the Control group, while Shionone reduced the increase in LPS-stimulated cells. The trends of Arg1, CD206, and p-STAT5/ STAT5 were increased in the Shionone groups, and the expression of ECM1 was decreased with the increasing dose of Shionone (Figures 5A,B).

### Protective Effect of Shionone on Sepsis-Induced Inflammation Was Mediated by Suppression of ECM1

**Figure 6:**
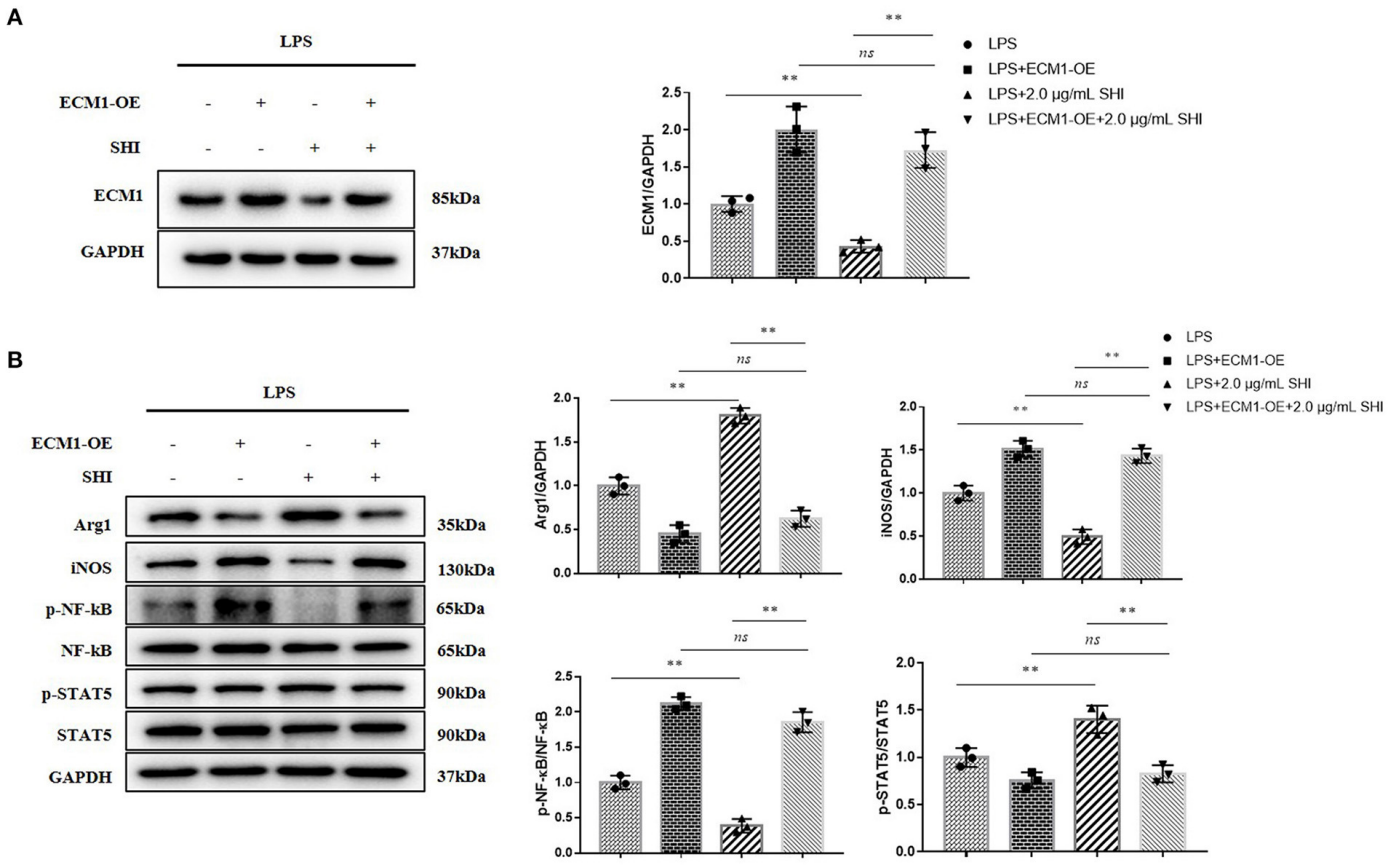
ECM1 overexpression reduces the inhibition of inflammation by Shionone. **(A)** Western blot analyses of expression of ECM1 and histogram of relative expression of each protein (*n* = 3). **(B)** Western blot analyses of expressions of Arg1, iNOS, p-NF-κB, NF-κB, p-STAT5, and STAT5 in LPS-stimulated RAW264.7 cells and histogram of relative expression of each protein (*n* = 3). Results are expressed as ± SD for independent experiments, **p < 0.01 are considered to be significantly different. ns = not statistically significant.

In the case of ECM1 being overexpressed, Arg1 and p-STAT5/STAT5 raised by Shionone were again reduced, and iNOS and p-NF-κB/NF-κB decreased by Shionone were again reduced (Figures 6A,B). This indicated that the protective effect of Shionone on sepsis-induced inflammation was mediated by suppression of ECM1.

## Discussion

The main pathological features of AKI are an inflammatory response and a rapid decline in renal function (21). Some studies have found that AKIis a major contributor to mortality in sepsis and is currently treated clinically with continuous renal replacement therapy (CRRT) (22–24). However, there are many problems with CRRT, such as inconsistent dose selection, filter changes, machine alarm pauses, and poor line patency (25–27). Therefore, we should first explore the possible pathogenesis of AKI and look for safer treatments.

AKI is usually associated with an elevation of pro-inflammatory cytokines, leading to leukocyte infiltration (1). In an *in vivo* model of AKI, serum levels of pro-inflammatory factors IL-6, IL-1β, IL-12, and TNF-α were increased in mice (Figures 2A–D), while those in Shionone-treated mice were reduced. As the basic cell type of the inflammatory response, macrophages are considered to be important conductors in AKI (28, 29). In patients with sepsis, large numbers of M1 macrophages migrate to the site of infection in the tissue, releasing pro-inflammatory factors and exacerbating tissue damage. Classical biomarkers of M1 macrophages include CD80, CD86 and CD16/32, and biomarkers of M2 macrophages include CD204, CD206, CD163, and CD23 (29, 30). As seen in Figures 1D,F, the increase in CD16/32 immunofluorescence intensity in kidney tissue suggested that M1 macrophage infiltration became more frequent, while Shionone treatment reduced M1 macrophages compared to the control group.

Macrophage polarization between classically activated M1 (anti-inflammatory) and alternatively activated M2 (pro-reparative) macrophages plays a complex role throughout AKI (30). GM-CSF treatment has been shown to reduce pro-inflammatory factors (20). LPS-stimulated STAT5 activation is largely dependent on GM-CSF and that phosphorylated STAT5 inhibits inflammatory cytokine transcription and promotes the expression of the M2 macrophage-associated gene Arg1 (31). Following alternative activation of M2 macrophages, Arg1 expression is tightly regulated in macrophages and therefore Arg1 is considered to be a marker of M2 macrophages (32). By examining protein levels in renal tissues, we hypothesized that the protective effect of Shionone on AKI mice may be *via* the GM-CSF/STAT5/Arg1 pathway to promote anti-inflammatory and tissue repair by M2 macrophages (Figures 2J,K).

ECM1 is highly expressed in macrophages infiltrated by inflammatory tissue and plays an important role in promoting M1 macrophage polarization to control inflammation and tissue repair (12). In *in vitro* cellular assays, we overexpressed ECM1 and found that the original promotion of Arg1 and p-STAT5 by Shionone was inhibited, while the inhibition of iNOS and p-NF-κB was blocked (Figure 6). Zhang's study found that deletion of ECM1 in macrophages resulted in the secretion of more GM-CSF, which in turn activated STAT5 to promote M2-type macrophages. Therefore, we infer that Shionone protects against to AKI by inhibiting ECM1, which activates GM-CSF/STAT5/Arg1 to promote M2 macrophages on the one hand, and reduces M1 macrophages on the other, which reduces inflammation and promotes tissue repair.

Therefore, in the present study, we used Shionone as an inhibitor of ECM1 to further confirm the relationship between macrophages and AKI. In an *in vivo* experiment, a significant decrease in the amount of pro-inflammatory factors secreted by M1 macrophages and an increase in anti-inflammatory factors secreted by M2 macrophages in mouse serum were observed in AKI mice with Shionone treatment. Meanwhile, Shionone concentrations with no toxic effect on cell viability were chosen to treat LPS-stimulated RAW264.7 cells and the assay results were consistent with *in vivo* experiments. After overexpression of ECM1, the protective effect of Shionone on LPS-stimulated RAW264.7 cells was found to be diminished.

However, there are limitations to this study. There are interactions between different intracellular signaling pathways and further studies are needed to investigate whether Shionone interferes with the GM-CSF/STAT5/Arg1 pathway from other signaling pathways. In addition, we believe that the inferior therapeutic effect of low-dose Shionone compared to DXM may be related to the low aspiration bioavailability of Shionone administered by gavage, and the same administration measures should be followed up for validation.

## Conclusion

In conclusion, the results of this study suggested that sepsis-induced AKI mice to produce M1 macrophages, which may contribute to the inflammatory response to AKI. By inhibiting ECM1 and activating the GM-CSF/STAT5/Arg1 pathway to promote alternative macrophage M2, Shionone reduces the inflammatory response to accelerate tissue repair and is able to attenuate AKI *in vitro* and *in vivo*. This study demonstrated that Shionone plays an important role in the treatment of AKI by regulating the polarization of macrophage, which provides a new idea to address AKI.

## Data Availability Statement

The raw data supporting the conclusions of this article will be made available by the authors, without undue reservation.

## Ethics Statement

The animal study was reviewed and approved by Suzhou Hospital of Integrated Traditional Chinese and Western Medicine. Written informed consent was obtained from the owners for the participation of their animals in this study.

## Author Contributions

BZ and YX: conceptualization, methodology, and writing—original draft. JZha: investigation and methodology. HJ: conceptualization. JZhu: methodology. HY: writing—original draft, investigation, and methodology. YQ: investigation, methodology, and writing—review and editing. AH: writing—review and editing. LX: writing—review and editing and investigation. YS and XW: investigation, supervision, project administration, formal analysis, conceptualization, and methodology. All authors contributed to the article and approved the submitted version.

## Funding

This work was supported by the Basic Research on Medical Health Application of Suzhou Science Program and Science Program of Suzhou Health Committee (No. SYSD2020234/GSWS2020117/KJXW2019072).

## Conflict of Interest

The authors declare that the research was conducted in the absence of any commercial or financial relationships that could be construed as a potential conflict of interest.

## Publisher's Note

All claims expressed in this article are solely those of the authors and do not necessarily represent those of their affiliated organizations, or those of the publisher, the editors and the reviewers. Any product that may be evaluated in this article, or claim that may be made by its manufacturer, is not guaranteed or endorsed by the publisher.
