# Supplementary material for: Shionone Attenuates Sepsis-Induced Acute Kidney Injury by Regulating Macrophage Polarization via the ECM1/STAT5 Pathway
Source: Front Med (Lausanne). 2022 Jan 24;8:796743. doi: 10.3389/fmed.2021.796743 (PMC8818860; doi:10.3389/fmed.2021.796743)
Supplement: Supplementary file 1 [file Presentation_1.PPTX]

## Slide 1
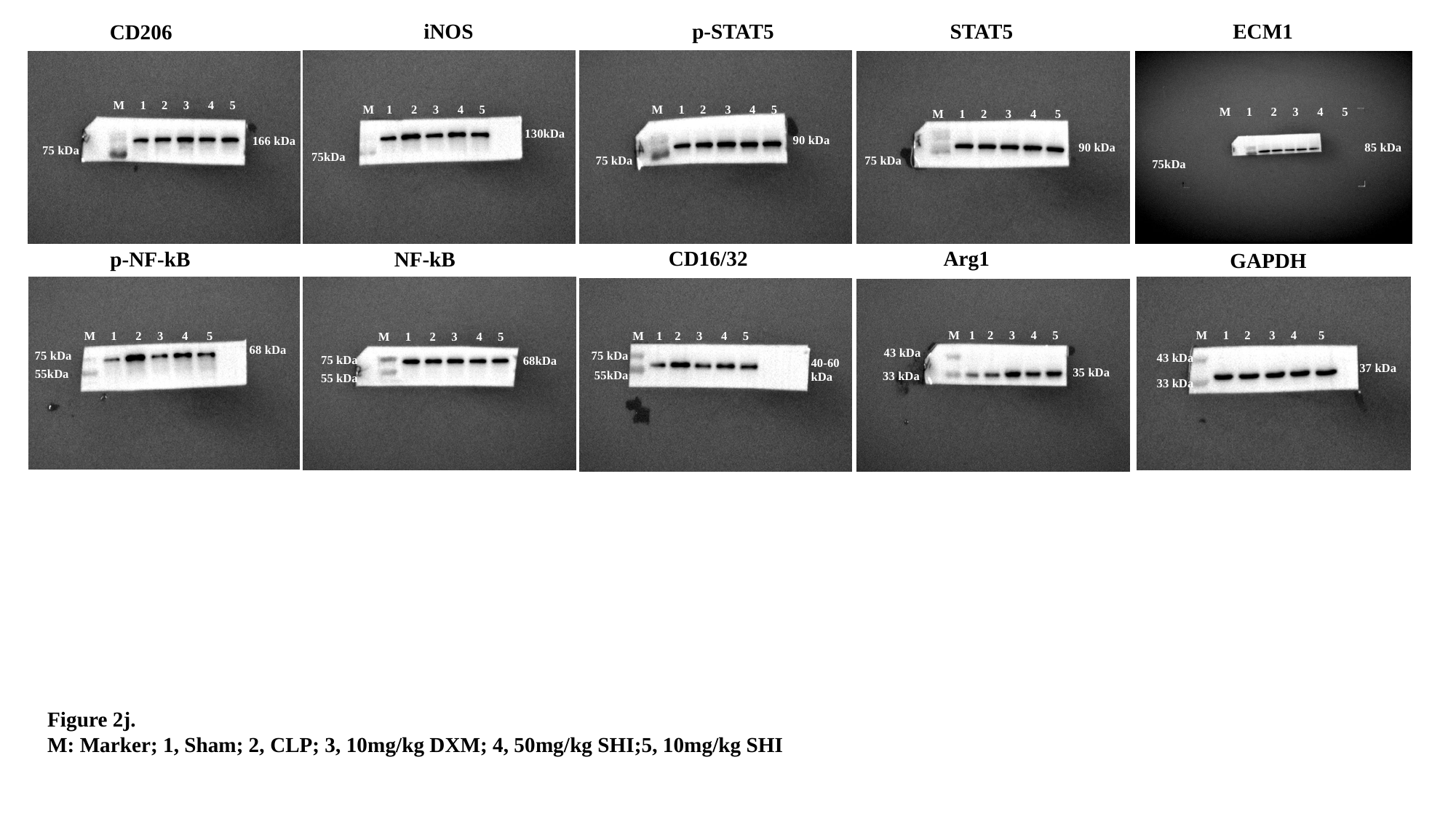

iNOS
p-STAT5
STAT5
ECM1
CD206
 M 1 2 3 4 5
 M 1 2 3 4 5
 M 1 2 3 4 5
 M 1 2 3 4 5
 M 1 2 3 4 5
130kDa
90 kDa
166 kDa
90 kDa
85 kDa
75 kDa
75kDa
75 kDa
75 kDa
75kDa
CD16/32
Arg1
p-NF-kB
NF-kB
GAPDH
M 1 2 3 4 5
 M 1 2 3 4 5
M 1 2 3 4 5
 M 1 2 3 4 5
 M 1 2 3 4 5
M 1 2 3 4 5
M 1 2 3 4 5
68 kDa
25 kDa
43 kDa
75 kDa
75 kDa
43 kDa
75 kDa
68kDa
40-60 kDa
40kDa
37 kDa
43 kDa
35 kDa
55kDa
55kDa
21 kDa
33 kDa
55 kDa
33 kDa
33 kDa
17 kDa
Figure 2j.
M: Marker; 1, Sham; 2, CLP; 3, 10mg/kg DXM; 4, 50mg/kg SHI;5, 10mg/kg SHI

## Slide 2
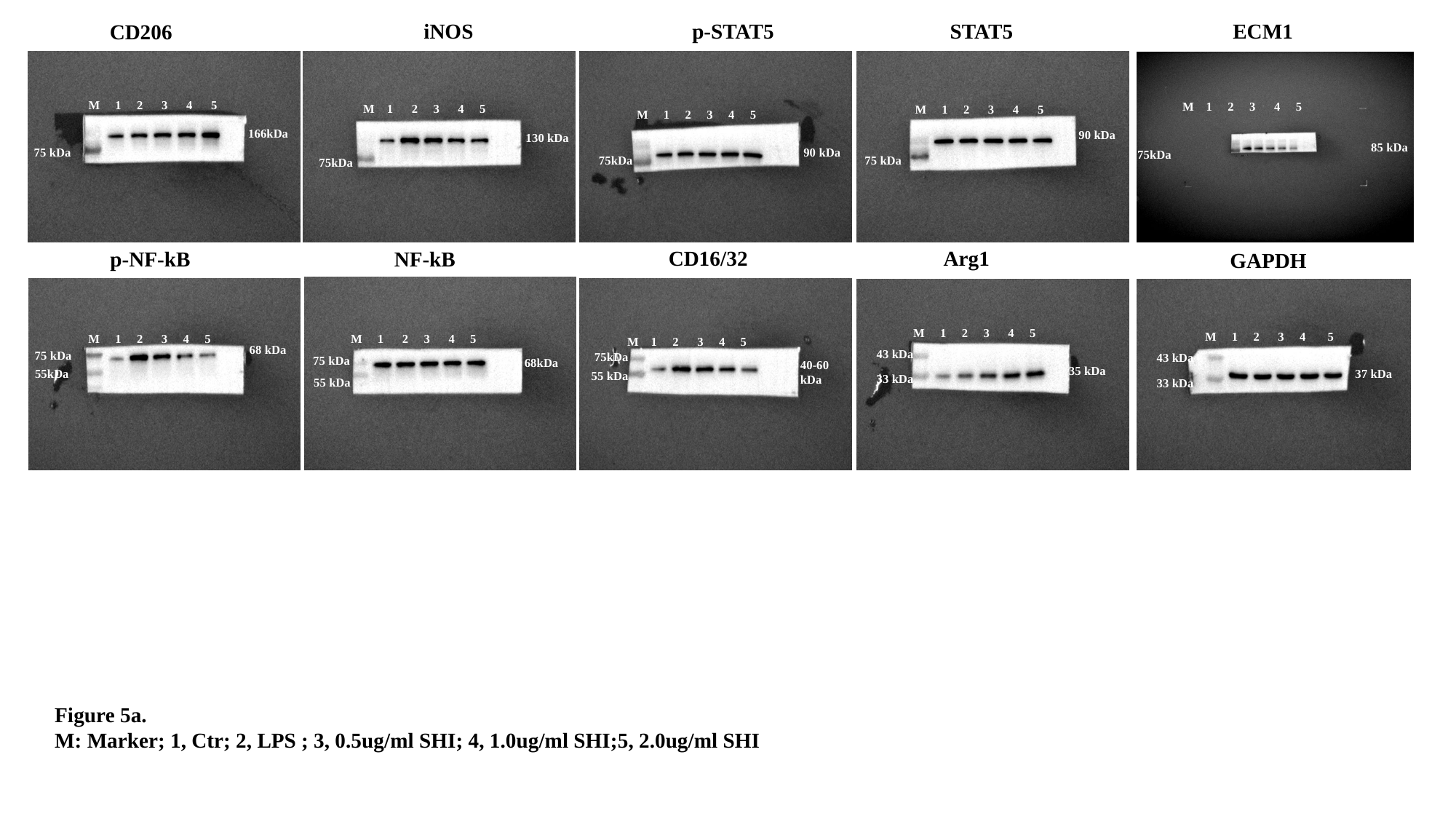

iNOS
p-STAT5
STAT5
ECM1
CD206
 M 1 2 3 4 5
 M 1 2 3 4 5
 M 1 2 3 4 5
 M 1 2 3 4 5
 M 1 2 3 4 5
 M 1 2 3 4 5
166kDa
90 kDa
130 kDa
85 kDa
85 kDa
90 kDa
75 kDa
75kDa
75kDa
75kDa
75 kDa
75kDa
CD16/32
Arg1
p-NF-kB
NF-kB
GAPDH
 M 1 2 3 4 5
M 1 2 3 4 5
 M 1 2 3 4 5
M 1 2 3 4 5
M 1 2 3 4 5
68 kDa
43 kDa
75 kDa
75kDa
43 kDa
75 kDa
68kDa
40-60 kDa
35 kDa
37 kDa
55kDa
55 kDa
33 kDa
55 kDa
33 kDa
Figure 5a.
M: Marker; 1, Ctr; 2, LPS ; 3, 0.5ug/ml SHI; 4, 1.0ug/ml SHI;5, 2.0ug/ml SHI

## Slide 3
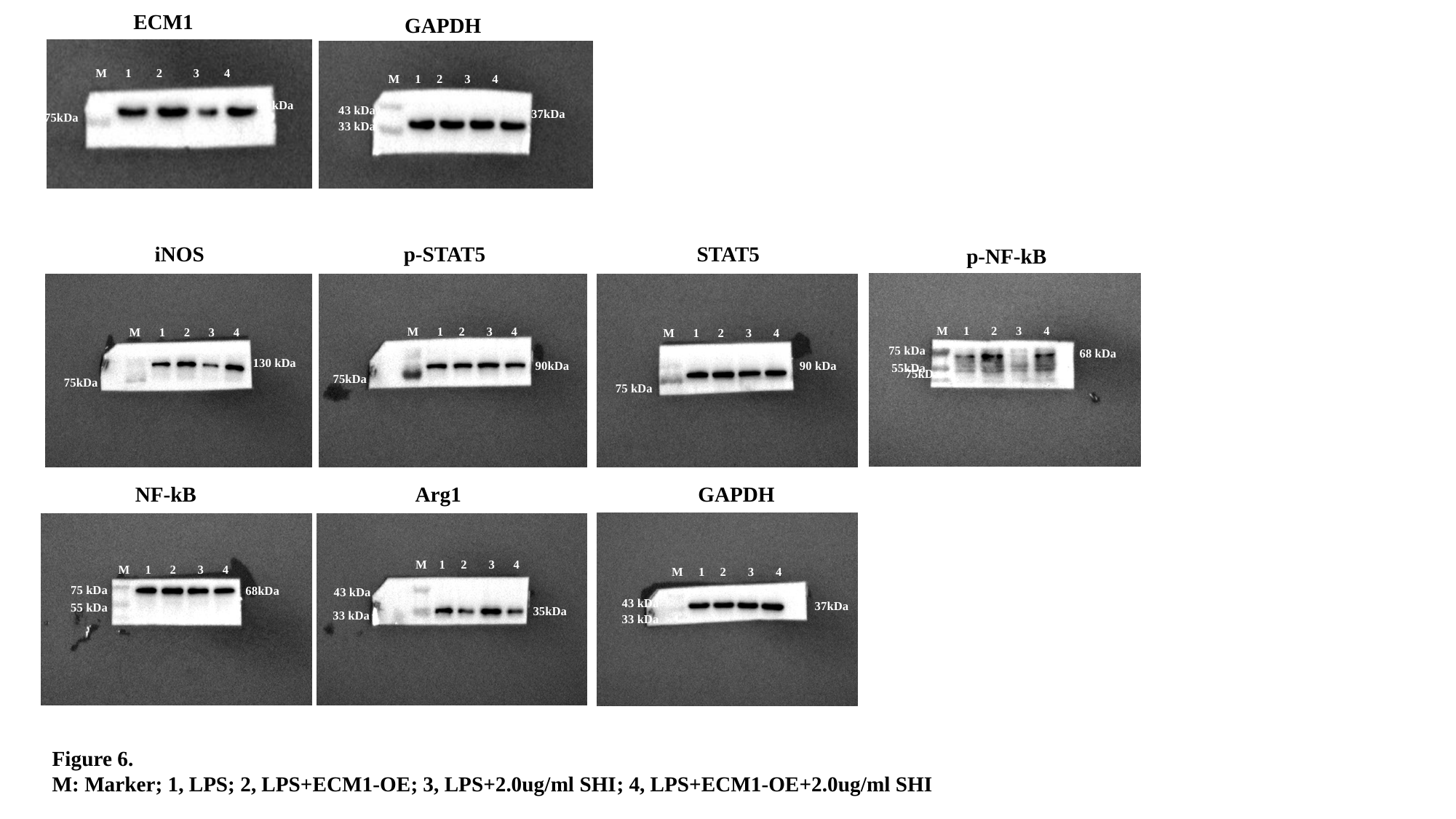

ECM1
GAPDH
M 1 2 3 4
M 1 2 3 4
85 kDa
43 kDa
37kDa
75kDa
33 kDa
 M 1 2
iNOS
p-STAT5
STAT5
p-NF-kB
M 1 2 3 4
M 1 2 3 4
 M 1 2 3 4
M 1 2 3 4
75 kDa
68 kDa
130 kDa
90kDa
90 kDa
55kDa
75kDa
75kDa
75kDa
75 kDa
NF-kB
Arg1
GAPDH
M 1 2 3 4
M 1 2 3 4
M 1 2 3 4
75 kDa
68kDa
43 kDa
43 kDa
43 kDa
37kDa
55 kDa
35kDa
33 kDa
33 kDa
33 kDa
Figure 6.
M: Marker; 1, LPS; 2, LPS+ECM1-OE; 3, LPS+2.0ug/ml SHI; 4, LPS+ECM1-OE+2.0ug/ml SHI
